# Bed Bugs and Infectious Disease: A Case for the Arboviruses

**DOI:** 10.1371/journal.ppat.1003462

**Published:** 2013-08-15

**Authors:** Zach N. Adelman, Dini M. Miller, Kevin M. Myles

**Affiliations:** Fralin Life Science Institute and Department of Entomology, Virginia Tech, Blacksburg, Virginia, United States of America; University of Florida, United States of America

## Bed Bugs and Infectious Diseases: 100 Years of Asking the Wrong Questions

Bed bug infestations (Cimicidae; *Cimex lectularius*) have been increasing worldwide over the last few decades [1], [2]. Several factors have been posited to explain this resurgence, including widespread insecticide resistance, human population growth, and increased international travel [1]. Clinically, reactions to bed bug bites vary from unapparent, to small (<5 mm) maculopapular lesions, to large wheals (2–6 cm); other reactions include bullous rashes, dermatitis, and asthma [1], [3]. However, in the developed world the psychological, social, and economic impacts of bed bugs may be the most troubling aspects of the resurgence [2]. While the bed bug invasion cuts across economic lines, those with sufficient resources are able to clear the infestations, while those without may have to live with their bed bugs into the foreseeable future [2], [4].

Bloodfeeding arthropods such as mosquitoes, ticks, fleas, kissing bugs, biting flies, and lice serve as biological vectors for human pathogens. Thus, it seems natural that bed bugs would also transmit infectious agents. However, more than 100 years of searching has produced little evidence to support this assumption. Comprehensive reviews examining bed bugs' ability to vector a wide range of high-profile human pathogens, such as HIV, MRSA, and hepatitis B, C, and E viruses, among others, have recently been published [1], [3]. Such experiments have so far failed to provide any convincing evidence of bed bugs in the transmission of these agents and thus will not be discussed here. Surprisingly, previous attempts to link bed bugs with disease transmission have largely omitted those viral pathogens known to have transmission cycles involving insect vectors. Thus, the purpose of this review is to refocus the attention of the research community on those pathogens most likely to be vectored by bed bug species given the appropriate ecological circumstances, and away from human pathogens with no previous history of transmission by insect vectors.

## Arbovirus Transmission Cycles

Nearly all arthropod-borne viruses (arboviruses) are maintained in nature by zoonotic transmission cycles involving an arthropod and nonhuman vertebrate host. Epidemics occur when transmission spills over to unintended hosts or the pathogen jumps into a new vector species [5], [6]. Thus despite over 100 years of searching, to conclude that bed bugs do not transmit infectious agents may still be premature, as the possibility of zoonotic transmission cycles involving bed bugs or related species has yet to be adequately and thoroughly investigated. As members of the family Cimicidae, the human bed bugs *C. lectularius* and *Cimex hemipterus* have many close relatives. Within this family are six recognized subfamilies, 22 genera, and at least 74 species; all are bloodsucking ectoparasites of bats and/or birds, including at least 14 additional species within the genus *Cimex* [7]. At least eight species from four genera are known to feed on humans when their bat/bird hosts nest in human dwellings, or when humans enter infested caves and/or disturb nests [4], [8]. Additionally, wild populations of the human bed bug *C. lectularius* are found in association with bat populations [7], [9], raising the possibility of a zoonotic transmission cycle involving this species. Gregarious bird or bat populations can easily number in the thousands or even millions [5]. The relatively short breeding times of bats and birds would ensure a sufficient supply of newly susceptible individuals, a requirement for sustaining zoonotic transmission cycles involving bat bugs, bird bugs, or even human bed bugs. Given that a number of arboviruses are known to be associated with bats, and the fact that bats have played an important role in the recent emergence of several viral human diseases [5], [10], the cimicid gut must regularly be exposed to viral pathogens following ingestion of bat blood meals. The question then becomes: can any of these viruses acquire adaptive mutations that would permit the use of a cimicid as a vector species, and has this already occurred?

## Bed Bugs and Alphavirus Transmission

**Figure 1:**
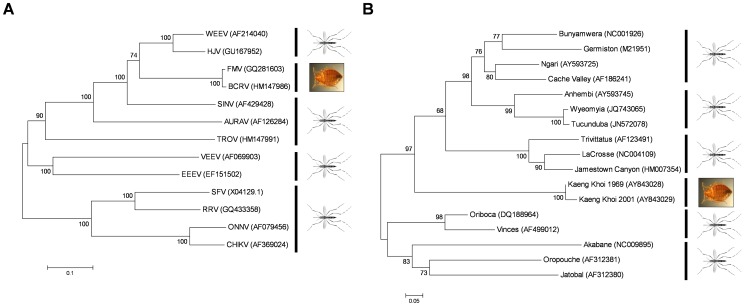
Evolutionary relationships of various mosquito-borne and cimicid-borne viruses. (**A**) Relationship of the structural proteins E2, 6K, and E1 from the alphaviruses chikungunya virus (CHIKV), o'nyong nyong virus (ONNV), Ross River virus (RRV), Semliki Forest virus (SFV), Trocara virus (TROV), Aura virus (AURAV), Sindbis virus (SINV), Buggy Creek virus (BCRV), Fort Morgan virus (FMV), Highland J virus (HJV), western equine encephalitis virus (WEEV), eastern equine encephalitis virus (EEEV), and Venezuelan equine encephalitis virus (VEEV). (**B**) Relationship among orthobunyaviruses following alignment of the first ∼100aa of the G2 glycoprotein. Both trees were produced in MEGA5 [31] using the neighbor-joining method; bootstrap support following 2,000 replicates is indicated.

In 1973 in Fort Morgan, Colorado, a new arbovirus related to western equine encephalitis virus (WEEV; family *Togaviridae*, genus *Alphavirus*) was repeatedly isolated from cliff swallows, house sparrows, and cliff swallow bugs (*Oeciacus vicarius*) [11]. Studies later confirmed that swallow bugs fed on birds infected with the virus, termed Fort Morgan virus (FMV), were infected at rates of 70–80%. Further, the infected swallow bugs were subsequently shown to be capable of transmitting the virus to susceptible uninfected birds [12]. In the decades since, it has become clear that FMV, and its close relative Buggy Creek virus (BCRV) [13], circulate extensively among cliff swallows throughout the western United States [14], [15]. These viruses are maintained in nature by a zoonotic transmission cycle between cliff swallows and swallow bugs; epizootic amplification also occurs when house sparrows invade cliff swallow nests and are fed upon by infected bugs [11], [16]–[18]. Members of the *Alphavirus* genus most closely related to FMV are usually transmitted in zoonotic cycles involving mosquitoes and birds (Figure 1A). Large populations of cliff swallows or house sparrows would be an attractive source of blood for the ornithophilic *Culex* spp. mosquitoes typically involved in the transmission of WEEV. In fact, WEEV was isolated from *Culex tarsalis* trapped in the vicinity of cliff swallow nests where FMV was first isolated [11]. Thus, the origin of FMV is best explained by a WEE-like virus originally transmitted between mosquitoes and birds adapting from using a mosquito vector to swallow bugs.

Another alphavirus, Tonate virus (TONV), was also repeatedly isolated from swallow bugs during the years 1974–1976 [19]. TONV is closely related to Venezuelan equine encephalitis virus (VEEV), and like its relative also causes fatal encephalitis in humans [20]. Unlike FMV, which primarily infects swallow bugs, TONV was found to infect *Oe. vicarius* at a rate of only 1–5%, much lower than its 45% infection rate for *Culex tarsalis* mosquitoes [19]. This may indicate that TONV has not yet adapted to use swallow bugs as a primary vector species. Whether TONV has continued to evolve towards the use of swallow bugs as vectors remains unclear, as the virus was not found in swallow bug populations in the year following 1976, and no further studies have been reported.

From these examples there is reason to suggest that other alphaviruses, such as eastern equine encephalitis virus (EEEV) or other members of the VEEV or WEEV complexes may have already adapted to cimicids, or could do so in the future. Despite a principle transmission cycle involving mosquitoes, EEEV has been isolated from naturally infected chicken mites [21]. This suggests that human bed bugs, which can also infest poultry farms [22], may be exposed to this virus as well. Although the mites were shown to be poor biological vectors, it is unknown how often such novel insect-virus pairings result in productive infections. Similarly, virtually nothing is known about the specific mutations that might be required to increase the fitness of an arboviral pathogen in a potentially novel insect vector. Although FMV has substantially diverged from WEEV, how many changes were initially required in the ancestral viral population to permit efficient infection, dissemination, and transmission by swallow bugs? The answers to these questions will provide substantial new insights into the potential for cimicids to serve as vectors of pathogenic alphaviruses under the appropriate ecological circumstances.

## Bed Bugs and Other Arboviruses

In 1970–1971, Kaeng Khoi virus (KKV; family *Bunyaviridiae*, genus *Orthobunyavirus*) was repeatedly isolated from bat bugs (*Cimex insuetus* and *Stricticimex parvus*) collected from several caves in Thailand [8]. KKV was not isolated from any of the bat ticks (*Ornithodorus hermsi*) also present in the caves, suggesting a true biological role for bat bugs in the transmission of the virus. The bat bugs aggressively fed on humans who entered the caves to mine bat guano; many such workers complained of illness after entering the caves for the first time and were found to be seropositive for KKV, suggesting true epizootic transmission was occurring [8]. Later work has since shown that KKV is widely present in bat populations over a large geographical area in southeast Asia [23]. While biological transmission of KKV by bat bugs has not been demonstrated experimentally, the phylogenetic classification of this virus suggests an insect vector; all closely related orthobunyaviruses have mosquito vectors (Figure 1B).

No isolations of flaviviruses have been reported from any cimicid species. Human bed bugs do not appear to be competent vectors of dengue viruses [24], and swallow bugs failed to transmit West Nile virus [25]. However, Entebbe bat virus (ENTV), Yokose virus (YOKV), and Sokuluk virus (SOKV) are all bat-associated viruses and group phylogenetically with mosquito-borne members of the genus *Flavivirus* [26]. Although no arthropod vector has ever been associated with these viruses, they retain the ability to replicate in arthropod cells, suggesting they may not have a strictly vertebrate host association [27].

## Virus Discovery and Bed Bugs

Despite the isolation of FMV, BCRV, TONV, and KKV from bat bugs and swallow bugs, no data exists as to whether these viruses can be transmitted by other cimicids, including human bed bugs. The identification of these viruses suggests that other cimicid populations may be involved in as yet undiscovered zoonotic transmission cycles with bats or birds. Virus discovery through deep sequencing can be used to identify new viral agents in an unbiased manner [28]. Such methods could be useful in identifying previously undiscovered enzootic transmission cycles involving cimicids, particularly those with the potential to spill over into human populations through the involvement of human bed bugs. Geographic locations with large populations of birds or bats supporting large populations of ectoparasites would be a logical starting point for such studies. More targeted efforts at virus discovery should focus on pathogens in the genera *Alphavirus*, *Flavivirus*, and *Orthobunyavirus*, due to their close associations with insect vectors and known propensity for causing disease in humans and/or animals.

## Concluding Remarks

Concluding that bed bugs do not transmit infectious agents may be premature, as most published studies that have investigated this question have focused on pathogens unlikely to be vectored by any bloodfeeding arthropod. While the ecological conditions associated with single-dwelling infestations are extremely unlikely to maintain arbovirus transmission cycles, the large and diverse number of cimicid species associated with highly gregarious bat or bird populations around the world (including wild *C. lectularius*) may well be supporting undiscovered enzootic transmission cycles. If this proves to be the case, the probability for spillover into human populations will increase as cimicid (including *C. lectularius* and *C. hemipterus*) populations continue to increase worldwide. Human populations most at risk would be those coexisting with long-term bed bug infestations such as those living refugee camps [29], homeless shelters [30], migrant worker camps, or similar situations; particularly those located in proximity to large bat/bird populations. While some dramatic exceptions exist, arbovirus infection typically presents with generic symptoms such as fever, rash, or joint pain. These nonspecific symptoms severely complicate accurate diagnosis, particularly in geographic locations where diseases like malaria and dengue are common. Our expectation is that the social and economic hardships that prevent these at-risk human populations from clearing infestations or relocating to bed bug-free environments also limit their access to medical diagnostics or care that might detect an emerging arbovirus. Thus, like virtually all vector-borne diseases worldwide, we expect that any bed bug–mediated disease transmission would be felt most heavily by the poor and underserved in the developing world. As noted previously [10], most of the >500 described arboviruses [24] were initially identified through intensive surveying of vertebrate or invertebrate populations, not in response to a specific human disease outbreak. Proactive identification and characterization of additional cimicid-based enzootic transmission cycles should be the focus of future studies, as these may serve as reservoirs of emerging viral pathogens. Similarly, reverse genetic experiments with FMV, KKV, and related viruses from known mosquito-based transmission cycles might be fruitful in elucidating the minimum number and type of amino acids changes necessary to alter the host range of these viruses, and hence the potential for human disease emergence.
